# 6‐Nitrodopamine Release From Mouse Seminal Vesicles Is Dependent on Endothelial Nitric Oxide Synthase (eNOS) Activation

**DOI:** 10.1002/prp2.70167

**Published:** 2025-08-22

**Authors:** José Britto‐Júnior, Pérola Rafaella Cedano Godoy, Denis Oliveira Lima, Valéria B. de Souza, André A. Schenka, Fernanda V. Mariano, Felipe Fernandes Jacintho, Idam Hermawan, Hiroaki Shimokawa, Masato Tsutsui, Silvana Chiavegatto, Edson Antunes, Gilberto De Nucci

**Affiliations:** ^1^ Department of Pharmacology, Faculty of Medical Sciences University of Campinas (UNICAMP) Campinas Brazil; ^2^ Department of Pathology, Faculty of Medical Science State University of Campinas (UNICAMP) Campinas Brazil; ^3^ Department of Pharmacology, Graduate School of Medicine University of the Ryukyus Okinawa Japan; ^4^ Graduate School International University of Health and Welfare Narita Japan; ^5^ Department of Pharmacology Institute of Biomedical Sciences (ICB), University of Sao Paulo (USP) São Paulo Brazil; ^6^ Department of Psychiatry Institute of Psychiatry (IPq), University of Sao Paulo Medical School (FMUSP) São Paulo Brazil

**Keywords:** ejaculation, epithelial cells, nitrocatecholamines, synergism

## Abstract

Human seminal vesicles present basal release of epithelium‐derived 6‐nitrodopamine (6‐ND) and this catecholamine potentiates noradrenaline‐induced contractions. Since nitric oxide synthase (NOS) activation is a determining factor involved in 6‐ND biosynthesis, this study aimed to investigate which NOS isoform is responsible for the 6‐ND release in mouse isolated seminal vesicles (MISV). Male control, eNOS^−/−^, NOS^−/−^, iNOS^−/−^, and e/n/iNOS^−/−^ mice were used. 6‐ND release was evaluated by liquid chromatography coupled to tandem mass spectrometry (LC–MS/MS). MISV contractility was assessed following electric‐field stimulation (EFS) or construction of concentration‐response curves to catecholamines. MISV from control, eNOS^−/−^, nNOS^−/−^, iNOS^−/−^, and e/n/iNOS^−/−^ mice exhibited basal release of 6‐ND, but 6‐ND release from eNOS^−/−^ and e/n/iNOS^−/−^ was significantly reduced compared to control and nNOS^−/−^ mice. Epithelium removal in MISV from control mice significantly reduced 6‐ND levels. EFS (2–32 Hz) induced frequency‐dependent MISV contractions in all groups, but the contractions from eNOS^−/−^ and e/n/iNOS^−/−^ mice were significantly decreased compared to control groups. In vitro l‐NAME treatment or epithelium removal significantly reduced EFS‐induced contractions. Pre‐incubation of MISV with 6‐ND (1–100 nM) significantly potentiated both EFS‐ and noradrenaline‐induced contractions, whereas pre‐incubation with noradrenaline, adrenaline, and dopamine did not affect the EFS‐induced responses. Immunohistochemistry and fluorescence in situ hybridization (FISH) assays revealed positivity for tyrosine hydroxylase and eNOS in the epithelium and endothelium of the seminal vesicles. In conclusion, MISV releases epithelium‐derived 6‐ND, and its biosynthesis involves eNOS activation. The finding that 6‐ND markedly amplified the noradrenaline‐induced contractions indicates that epithelium‐derived 6‐ND acts as a major modulator of MISV contractility.

## Graphical abstract

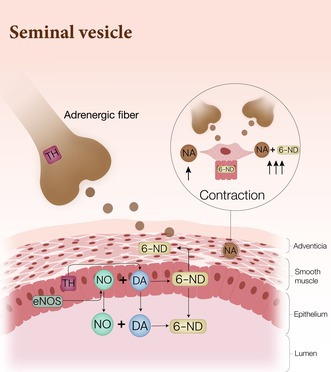

## 1 Introduction

Endothelium‐derived 6‐nitrodopamine (6‐ND) exerts potent physiological effects, including positive chronotropic and inotropic effects in rats, and marked vasodilation in different animal species [1]. In the heart, 6‐ND synthesis is dependent on endothelial nitric oxide synthase (eNOS) activation, since it is significantly decreased in eNOS^−/−^ mice [2]. Outside the cardiovascular system, 6‐ND has been identified in human [3], rat [4] and mouse [5] isolated vas deferens, where it potentiates the contractions induced by the classical catecholamines noradrenaline, adrenaline, and dopamine [6]. In the vas deferens, 6‐ND synthesis is dependent on the neuronal nitric oxide synthase (nNOS) activation, as demonstrated in nNOS^−/−^ mice [5].

Seminal vesicles play an important role in the first phase of the ejaculatory process, that is, emission, which consists of the ejection into the prostatic urethra of spermatozoa mixed with fluids secreted by accessory sexual glands [7]. Besides the contractile role in the emission process, seminal vesicles produce the seminal fluid [8]. In humans, seminal vesicle secretions represent 80% of semen volume and are essential for the normal ejaculate composition [9]. Seminal vesicles are supplied by cholinergic [10], adrenergic [11], and non‐adrenergic non‐cholinergic (NANC) innervation [12], but the sympathetic innervation originating from the hypogastric nerve plays a major role in the seminal vesicle contractility via α_1_‐adrenoceptors [13, 14]. Besides the classical catecholamines noradrenaline, adrenaline, and dopamine [15, 16, 17], human [18] and rat [19] seminal vesicles release 6‐ND, as measured by liquid chromatography coupled to tandem mass spectrometry (LC–MS/MS).

Because NOS activation is crucial for 6‐ND biosynthesis in different tissues, this study investigated which NOS isoform is determinant for the 6‐ND biosynthesis in the mouse seminal vesicles. This was addressed by using knockout (KO) mouse models for each NOS isoform, namely, eNOS, nNOS, and iNOS, as well as the triple‐n/i/eNOS‐KO mice. This study also explored how 6‐ND modulates seminal vesicle contractility, which was achieved by evaluating the contractile responses induced by both neurogenic stimulation (electric‐field stimulation) and noradrenaline, along with molecular assays (immunohistochemistry and fluorescence in situ hybridization) for tyrosine hydroxylase (TH), a rate‐limiting enzyme involved in catecholamine synthesis.

## 2 Methods

### 2.1 Animals

The investigation was performed in male iNOS^−/−^ (C57BL/6129P2‐Nos2tm1Lau/J), eNOS^−/−^ (C57BL/6129P2‐Nos3tm1Unc/J), nNOS^−/−^ (C57BL/6), and triple knockout (e/n/iNOS^−/−^; 18) mice in comparison with wild‐type (WT; control) C57BL/6J mice matched by age (10–15 weeks age). The control (WT), iNOS^−/−^ and eNOS^−/−^ mice were acquired from Jackson Laboratory (Maine, USA), with breeding colonies established by the Multidisciplinary Center for Biological Research in the Field of Laboratory Animal Science (CEMIB) of UNICAMP (São Paulo, Brazil). The nNOS^−/−^ mouse were obtained from Prof. S. Chiavegatto at IMT‐FMUSP (São Paulo, Brazil). The triple knockout (e/n/iNOS^−/−^) mice were provided by Prof. M. Tsutsui from the University of the Ryukyus (Okinawa, Japan), with the breeding colony established at CEMIB/UNICAMP. All scientific procedures were reviewed by the UNICAMP Ethics Committee for Animal Use (CEUA; Protocol No. 5959‐1/2022 and 6337‐1/2023) and followed the CONCEA [20] and the ARRIVE guidelines [21]. Animals were kept under controlled room temperature (22°C–24°C), a 12‐h light cycle, and food and water ad libitum.

### 2.2 Isolation of Mouse Seminal Vesicles

Euthanasia was carried out using an isoflurane overdose (> 5%) until respiration ceased, typically within 1 min. Exsanguination was then performed. Following euthanasia, the seminal vesicles from each mouse were removed and kept in Krebs–Henseleit solution (KHS; in mM: NaCl 118, KCl 4.7, CaCl_2_ 2.5, MgSO_4_ 1.7, NaHCO_3_ 24.9, KH_2_PO_4_ 1.2, dextrose 11) at 8°C. For functional assay experiments, each seminal vesicle was carefully dissected and mounted vertically in a glass bath filled with 10 mL containing KHS (pH 7.4). The vesicles were secured using fine cotton thread, with one end tied to a metal pin and the other end connected to an isometric force transducer. The bathing solution was continuously aerated with 95% O_2_ and 5% CO_2_ and the temperature (37°C) kept constant by PolyScience (Illinois, USA) heated circulator. Following a 45‐min stabilization period, the mouse isolated seminal vesicles were contracted with a single concentration of potassium hydrochloride (KCl, 80 mM) to verify the tissue viability.

### 2.3 Measurement of 6‐ND and Other Catecholamines (Noradrenaline, Adrenaline and Dopamine) in Isolated Seminal Vesicles by LC–MS/MS

Isolated seminal vesicles from two mice (a total of four vesicles) were suspended separately in 3‐mL glass baths containing KHS with ascorbic acid (3 mM), continuously gassed with a mixture of 95% O_2_ and 5% CO_2_ at 37°C for 30 min. In some protocols, epithelial cells were mechanically removed by gently rubbing the seminal vesicle with metal forceps. After incubation, a 2‐mL aliquot of the supernatant was transferred to black microtubes and stored at −20°C until analysis by LC–MS/MS [21, 22]. Results are expressed as *x*/*y*, where *x* represents the number of animals used and y the number of samples analyzed by LC–MS/MS.

The extraction and quantification of 6‐ND in KHS were performed as reported previously [21, 22]. Briefly, 6‐ND was extracted from 1 mL of KHS by solid phase extraction (SPE). Calibrators and quality controls (QCs) prepared in blank KHS and KHS samples obtained from mouse seminal vesicles were spiked with 50 μL of the internal standard (IS) solution (6‐ND‐d_4_, 100 ng/mL). Extraction cartridges were conditioned with 1 mL of methanol and then equilibrated with 2 mL of deionized water. The samples were transferred to the extraction cartridges and washed three times with deionized water. The samples were eluted with 0.9 mL methanol/deionized water (90/10, v/v) plus 0.1% formic acid and followed by evaporation under N_2_ flow at 50°C. The dry residues were dissolved with 100 μL acetonitrile/deionized water (50/50, v/v) plus 0.1% formic acid, transferred to vials, and submitted to chromatographic analysis. The separation of 6‐ND was performed on a 150 mm × 3.0 mm Shim‐pack GIST‐HP C18 column, 3‐μm particle size (Shimadzu, Duisburg, Germany) held at 65°C. A 75% of mobile phase A consisting of deionized water with 0.1% formic acid (v/v) and 25% of mobile phase B consisting of acetonitrile/deionized water (90/10, v/v) plus 0.1% formic acid at a flow rate of 350 μL/min in an isocratic mode were used. The detection of 6‐ND and IS was carried out by a LC–MS‐8060 triple quadrupole mass spectrometer (MS/MS) (Shimadzu, Kyoto), operating in positive ionization mode. The analyses were performed in the multiple reaction monitoring (MRM) mode. The protonated ions [M + H]^+^ and their respective ion products monitored were 199.10 > 181.95 and 203.10 > 186.00 for 6‐ND and 6‐ND‐d4, respectively. In contrast to the original method, two transitions for each analyte were employed to enhance selectivity; one transition was employed as the quantifier transition (199.10 > 181.95) and the other was employed as the qualifier transition (199.10 > 136.00).

### 2.4 Effect of Electric‐Field Stimulation (EFS) in Mouse Isolated Seminal Vesicles

Mouse seminal vesicle strips were submitted to EFS generated by a Grass S88 stimulator (Astro‐Medical, Industrial Park, RI, USA). The voltage and frequencies used were 60 V for 20 s, at 2–16 Hz with square‐wave pulses, a pulse width of 0.3 ms, and a delay of 0.1 ms. EFS‐induced seminal vesicle contractions were evaluated in the absence and the presence of the non‐selective NOS inhibitor l‐NAME (100 μM, 30 min), as well as in preparations with denuded epithelial cells. In separate protocols, the preparations were pre‐incubated (30 min) with 6‐ND, noradrenaline, adrenaline, or dopamine (100 nM each), followed by the application of EFS. Data of EFS‐induced contractions were expressed in mN.

### 2.5 Concentration–Response Curves to Catecholamines in Mouse Isolated Seminal Vesicle

Cumulative concentration–response curves in isolated mouse seminal vesicle strips were generated for 6‐ND (10 nM–300 μM), noradrenaline (10 nM–300 μM), adrenaline (10 nM–300 μM), and dopamine (10 nM–300 μM). Additionally, to assess the interaction between 6‐ND and noradrenaline on seminal vesicle contractility, concentration–response curves to noradrenaline (10 nM–300 μM) were performed in the absence and presence of 6‐ND (1–100 nM, pre‐incubated for 30 min).

### 2.6 Immunohistochemistry

The mouse seminal vesicle samples (*n* = 5) were fixed in 10% neutral phosphate‐buffered formalin, embedded in paraffin, and sectioned to a thickness of 4 μm. The sections were deparaffinized in xylene and hydrated using a graded ethanol series. To block endogenous peroxidase activity, the sections were treated with 3% H_2_O_2_ for 10 min. Antigen retrieval was fulfilled by heating the section in citrate buffer at pH 6.0 (for anti‐tyrosine hydroxylase [TH]) or Epitope Retrieval Solution (for anti‐eNOS) (pH 9.0, Novocastra; Leyca biosystems), using a steamer set for 20 min (at approximately 95°C). Slides were incubated with a chicken polyclonal anti‐TH (catalog code: ab76442; dilution: 1:500 diluted in PBS; Abcam) and rabbit polyclonal anti‐eNOS antibodies (catalog code: ab5589, 1:50 diluted in PBS; Abcam) for 2 h at room temperature (*r*/*t*). Tissue sections receiving the chicken anti‐TH antibody only were sequentially incubated with a goat anti‐chicken gamma immunoglobulin IgG (ab150169; 1:500 diluted in PBS; Abcam) and rabbit anti‐goat IgG (AP106P; 1:250 diluted in PBS, Merck/Sigma) for 1 h each. Following incubation with the primary anti‐eNOS antibody or tertiary antibodies of the TH protocol, these samples were incubated with the NovoLink Max Polymer Detection System (Novocastra/Leica Biosystems) for TH or the goat pAB to Rb IgG HRP polymer (ab14880; Abcam) for eNOS, according to the manufacturer's instructions. The immunoreaction was performed with diaminobenzidine (liquid DAB, DakoCytomation) as chromogen, followed by Harris's hematoxylin counterstaining. After this, the sections were coverslipped with Entellan mounting medium. Negative controls consisted of the omission of the primary antibody and incubation with the primary antibody diluents to identify any unspecific background staining. The slides were examined using a trinocular Eclipse 50i microscope (Nikon, Tokyo, Japan) coupled to a 10MP CMOS digital camera (AmScope, EUA).

### 2.7 Fluorescence In Situ Hybridization (FISH) Assay for Tyrosine Hydroxylase

To confirm and validate TH protein expression and its topography, we further investigated TH mRNA expression in the same samples using a FISH assay. Briefly, sections from two mouse seminal vesicles were deparaffinized with xylene and rehydrated in graded alcohols for 5 min each. Then, the slides were incubated in a 0.2 N HCl solution for 20 min, and subsequently treated with a citrate pH 6.0 buffer (ZytoVision kit, catalog code Z‐2028‐20, Germany) at 80°C for 1 h. After this, they were incubated with pepsin for 8 min at room temperature. The slides were washed with 2 × SSC (ZytoVision kit, catalog code Z‐2028‐20, Germany). They were subsequently submitted to a sequence of ethanols (75%, 80%, and 100% ethanol for 2 min each), and then air dried. The slides were further incubated with a TH probe (at a concentration of 10 μM, in RNAse‐free water) for 10 min at 75°C and overnight in a Dako Hybridizer (Dako, Denmark) at 37°C. The TH probe sequence was as follows: 5′‐AACCGCGGGGACATGATGGCCT‐3′ (RNA Tm = 77.8°C) (Batch: WD11655417; Sigma/Merck, Germany). The probe was labeled with fluorescein 6‐FAM in the 5′ region. The next day, the slides were placed in a UREA/0.1 × SSC solution at 45°C for 30 min, and then they were washed with a 2 × SSC solution for 2 min. After this, the slides were dehydrated in 75%, 85%, and 100% ethanols for 2 min each, and air dried. Finally, the slides were mounted with 15 μL of a DAPI‐containing mounting medium (from the ZytoVision kit) and cover slipped (the cover slip being sealed with a Fixogum Rubber Cement, from Marabu, Germany). Negative controls consisted of the omission of the probe and were performed in all FISH assays (one negative control per section) to control any significant autofluorescence. All FISH slides were examined and photomicrographed using a trinocular DM4000 B LED microscope (Leica Microsystems, Wetzlar, Germany) coupled to a 1.4 MP DFC 310 FX camera (Leica, Switzerland).

### 2.8 Western Blot Analysis

Frozen mouse seminal vesicle tissues were pulverized and homogenized in cold RIPA lysis buffer supplemented with protease and phosphatase inhibitors (1 mM PMSF, 1 mM Na₃VO₄, and protease inhibitor cocktail). Protein extracts (30 μg) were separated via SDS‐PAGE and transferred to PVDF membranes. After blocking with 5% albumin in Tris buffer, membranes were incubated overnight at 4°C with primary antibodies against phospho‐eNOS at Ser 1177 (Cell Signaling; #9571, 1:500) or beta‐actin (Thermo Fisher; #MA5‐15739‐HRP, 1:25 000). After washing, membranes were incubated with HRP‐conjugated secondary antibodies and developed using an ECL detection system. Signal detection was performed with a chemiluminescent imager, and band intensities were quantified using ImageJ software.

### 2.9 Data Analysis

The results are reported as the mean ± standard error of the mean (SEM) from *n* experiments. Student's two‐tailed unpaired *t*‐test was used to analyze differences between two groups. For comparisons involving more than two groups, one‐way ANOVA followed by the Newman–Keuls post‐test was performed. A *p‐*value of less than 5% was taken as statistically significant. Nonlinear regression analysis to determine the pEC_50_ was performed using GraphPad Prism (GraphPad Software, version 10, San Diego, California, USA) with the constraint that *F* = 0. All concentration–response data were evaluated for fit to a logistic function in the form: *E* = *E*_max_/([1 + (10^*c*^/10^*x*^)^*n*^] + *F*), where *E* represents the contractile response increase induced by the agonist, *E*_max_ is the maximum agonist effect, *c* is the logarithm of the agonist concentration that produces 50% of *E*_max_, *x* is the logarithm of the drug concentration, *n* is a curve fitting parameter that defines the slope of the concentration–response curve, and *F* is the response observed in the absence of the drug.

## 3 Results

### 3.1 Basal Release of 6‐ND From Isolated Seminal Vesicles in NOS Gene Knockout Mice

**FIGURE 1:**
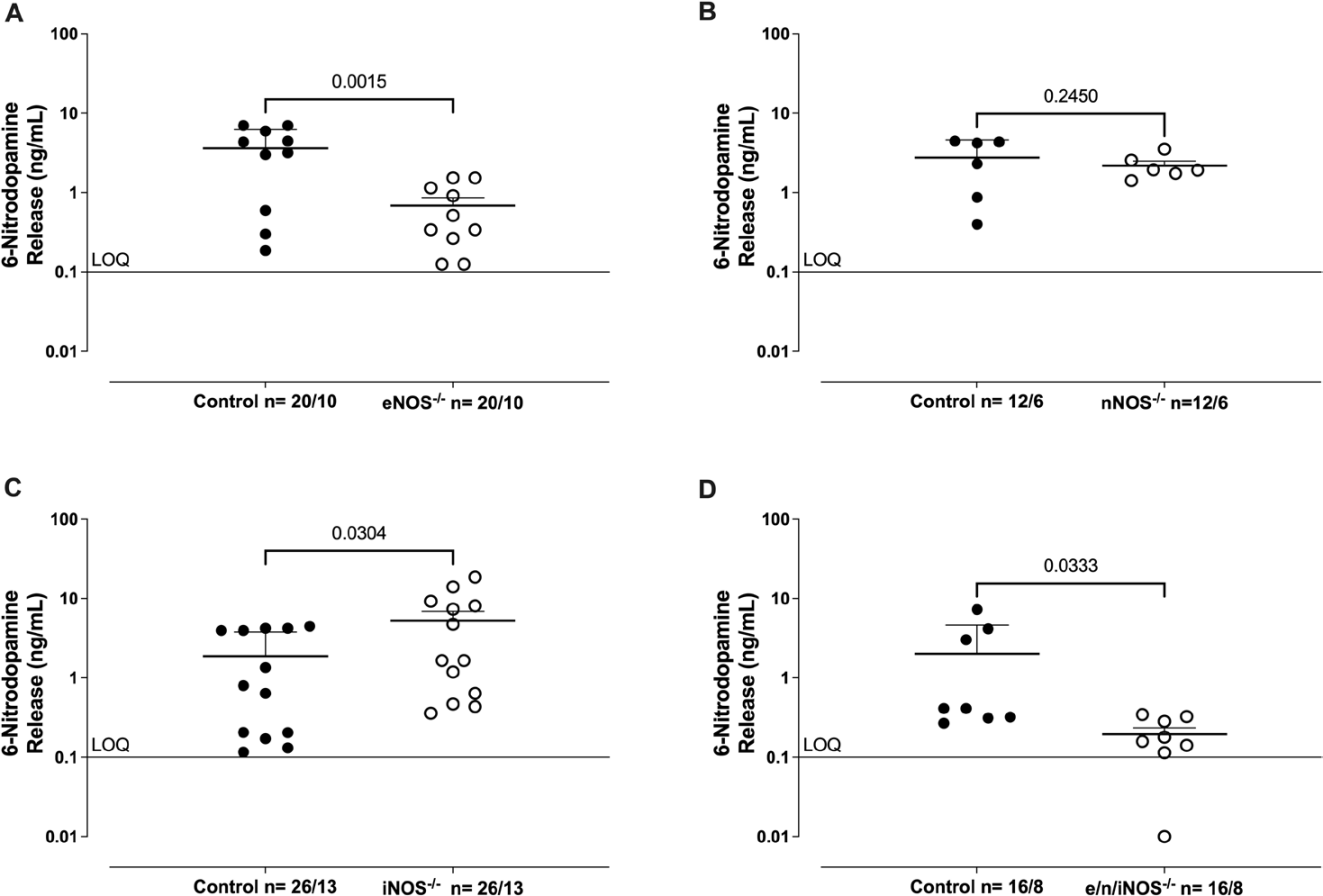
Basal release of 6‐nitrodopamine (6‐ND) from seminal vesicles obtained from control (wild‐type), eNOS^−/−^, nNOS^−/−^, iNOS^−/−^, and e/n/iNOS^−/−^ mice. Release of 6‐ND (A–D) was detected in Krebs–Henseleit's solution (KHS) by LC–MS/MS. The limit of quantification (LOQ) was 0.1 ng/mL. The number of seminal vesicle (*n*) is expressed as *x*/*y*, where *x* represents the number of animals and *y* the number of samples analyzed by LC–MS/MS. Data are shown as mean ± SEM and represent the value obtained from two pairs of seminal vesicles obtained from two mice.

Isolated seminal vesicles from control (Figure 1A–D), eNOS^−/−^ (Figure 1A), nNOS^−/−^ (Figure 1B), iNOS^−/−^ (Figure 1C), and e/n/iNOS^−/−^ mice (Figure 1D) exhibited basal release of 6‐ND, as detected by LC–MS/MS. In the eNOS^−/−^ (Figure 1A) and e/n/iNOS^−/−^ groups (Figure 1D), 6‐ND levels were significantly reduced compared to their respective control groups. In seminal vesicles from nNOS^−/−^ mice (Figure 1B), no significant differences in 6‐ND levels were observed compared to controls. However, in the iNOS^−/−^ group (Figure 1C), 6‐ND levels were significantly increased compared to control animals (*p* < 0.05). The levels of dopamine, noradrenaline, and adrenaline were below the limit of quantitation (LOQ; 0.1 ng/mL) in all samples.

### 3.2 EFS‐Induced Mouse Isolated Seminal Vesicle Contractions

**FIGURE 2:**
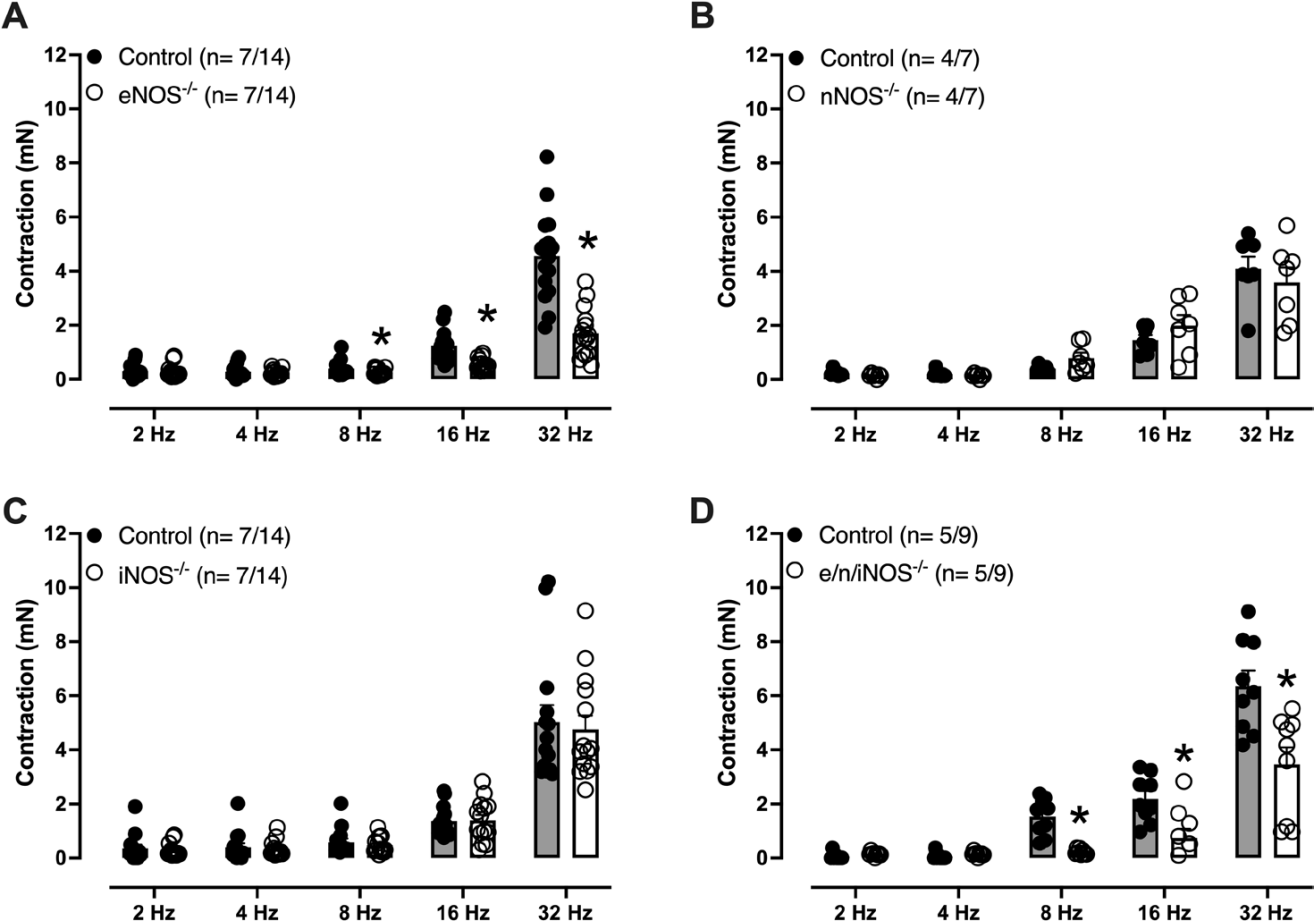
Isolated seminal vesicle contractions induced by electric‐field stimulation (EFS, 2–32 Hz) in isolated seminal vesicle from control (wild type), eNOS^−/−^, nNOS^−/−^, iNOS^−/−^, and e/n/iNOS^−/−^ mice. EFS‐induced seminal vesicle contractions in eNOS^−/−^ (A) and e/n/iNOS mice (D) are significantly reduced compared with control mice. In nNOS^−/−^ (B) and iNOS^−/−^ groups (C), the amplitude of EFS‐induced contractions did not significantly differ from control animals. Data are shown as mean ± SEM. The number of seminal vesicle (*n*) is expressed as *x*/*y*, where *x* represents the number of animals and *y* the number of seminal vesicles. *p < 0.05 compared versus respective control group.

EFS (2–32 Hz) induced frequency‐dependent contractions in mouse isolated seminal vesicles in all groups (Figure 2A–D). However, in eNOS^−/−^ (Figure 2A) and e/n/iNOS^−/−^ (Figure 2D) groups, EFS‐induced contractions were significantly reduced compared to control mice. In contrast, EFS‐induced contractions remained unchanged in seminal vesicles from nNOS^−/−^ (Figure 2B) and iNOS^−/−^ (Figure 2C) compared to 3 control mice.

### 3.3 Effect of l‐NAME on the Contractions Induced by EFS

**FIGURE 3:**
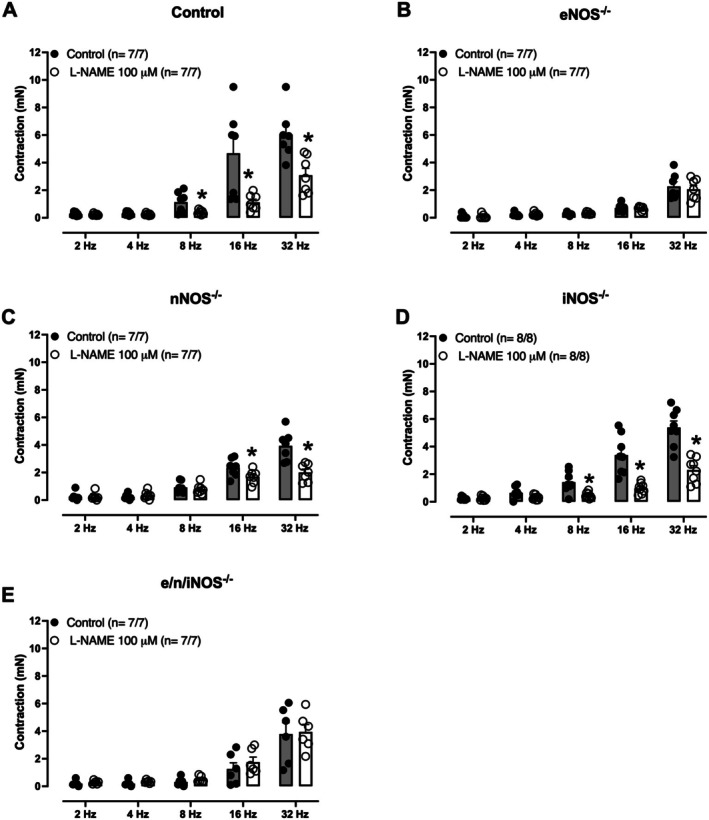
Effect of l‐NAME on the electric‐field stimulation (EFS, 2–32 Hz)‐induced seminal vesicle contractions from control, eNOS^−/−^, nNOS^−/−^, iNOS^−/−^ and e/n/iNOS^−/−^ mice. Pre‐incubation (30 min) with l‐NAME (100 μM) caused significant reductions of EFS‐induced contractions in control (A), nNOS^−/−^ (C) and iNOS^−/−^ groups (D) without affecting the responses in eNOS^−/−^ (B) and e/n/iNOS groups (E). Data are shown as mean ± SEM. *p < 0.05 compared with the respective control group. The number of seminal vesicles (*n*) is expressed as *x*/*y*, where *x* represents the number of animals and *y* the number of seminal vesicles.

As mentioned above, EFS (2–32 Hz) induced frequency‐dependent contractions of the mouse isolated seminal vesicle in all groups. Pre‐incubation (30 min) with l‐NAME (100 μM) significantly reduced the EFS‐induced seminal vesicle contractions in control (Figure 3A), nNOS^−/−^ (Figure 3C), and iNOS^−/−^ mice (Figure 3D), without affecting the responses in eNOS^−/−^ (Figure 3B) or e/n/iNOS^−/−^ mice (Figure 3E).

### 3.4 Effect of Epithelium Removal on Basal Release of 6‐ND and on EFS‐Induced Contractions

**FIGURE 4:**
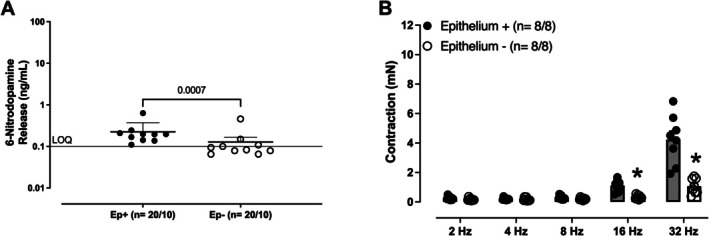
The basal release of 6‐ND and electric‐field stimulation (EFS, 2–32 Hz)‐ induced contractions in isolated seminal vesicle from control mice. The basal release of 6‐ND (A) was evaluated in intact (Ep+) and denuded epithelium (Ep−) preparations. The mechanical removal of the epithelium (B) caused a significant reduction in EFS‐induced contractions compared to intact epithelium preparations. Data are shown as mean ± SEM. The number of seminal vesicles analyzed is expressed as *x*/*y*, where *x* is the number of animals and *y* is the number of samples analyzed by LC–MS/MS or EFS‐induced contractions. *p < 0.05 compared with the respective control group.

Mechanical removal of seminal vesicle epithelium in control mice caused a significant decrease in both the basal release of 6‐ND (Figure 4A) and the EFS‐induced contractions (Figure 4B).

### 3.5 Potentiation of EFS‐Induced Seminal Vesicle Contractions by 6‐ND

**FIGURE 5:**
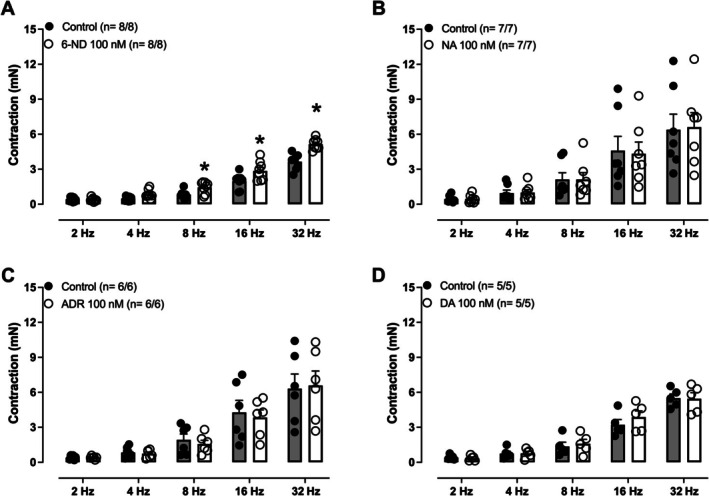
Effect of pre‐incubation with 6‐nitrodopamine (6‐ND), noradrenaline (NA), adrenaline (ADR) and dopamine (DA) on electric‐field stimulation (EFS)‐induced mouse seminal vesicle contractions in control mice. 6‐Nitrodopamine (6‐ND, 100 nM) significantly potentiated the EFS‐induced contractions (A) whereas noradrenaline [NA, 100 nM, (B)], adrenaline [ADR; 100 nM, (C)] and dopamine [DA; 100 nM, (D)] failed to affect the EFS‐induced contractions. The number of seminal vesicles analyzed is expressed as *x*/*y*, where *x* is the number of animals and *y* the number of seminal vesicles. *p < 0.05 (A) compared with the respective control group.

Pre‐incubation of seminal vesicle with 6‐ND (100 nM) significantly potentiated the contractions induced by EFS, particularly at the frequencies of 8, 16, and 32 Hz (Figure 5A). In contrast, pre‐incubation with noradrenaline (100 nM; Figure 5B), adrenaline (100 nM; Figure 5C), or dopamine (100 nM; Figure 5D) did not significantly affect EFS‐induced contractions.

### 3.6 Contractions of Mouse Isolated Seminal Vesicle by 6‐ND, Dopamine, Noradrenaline, and Adrenaline

**FIGURE 6:**
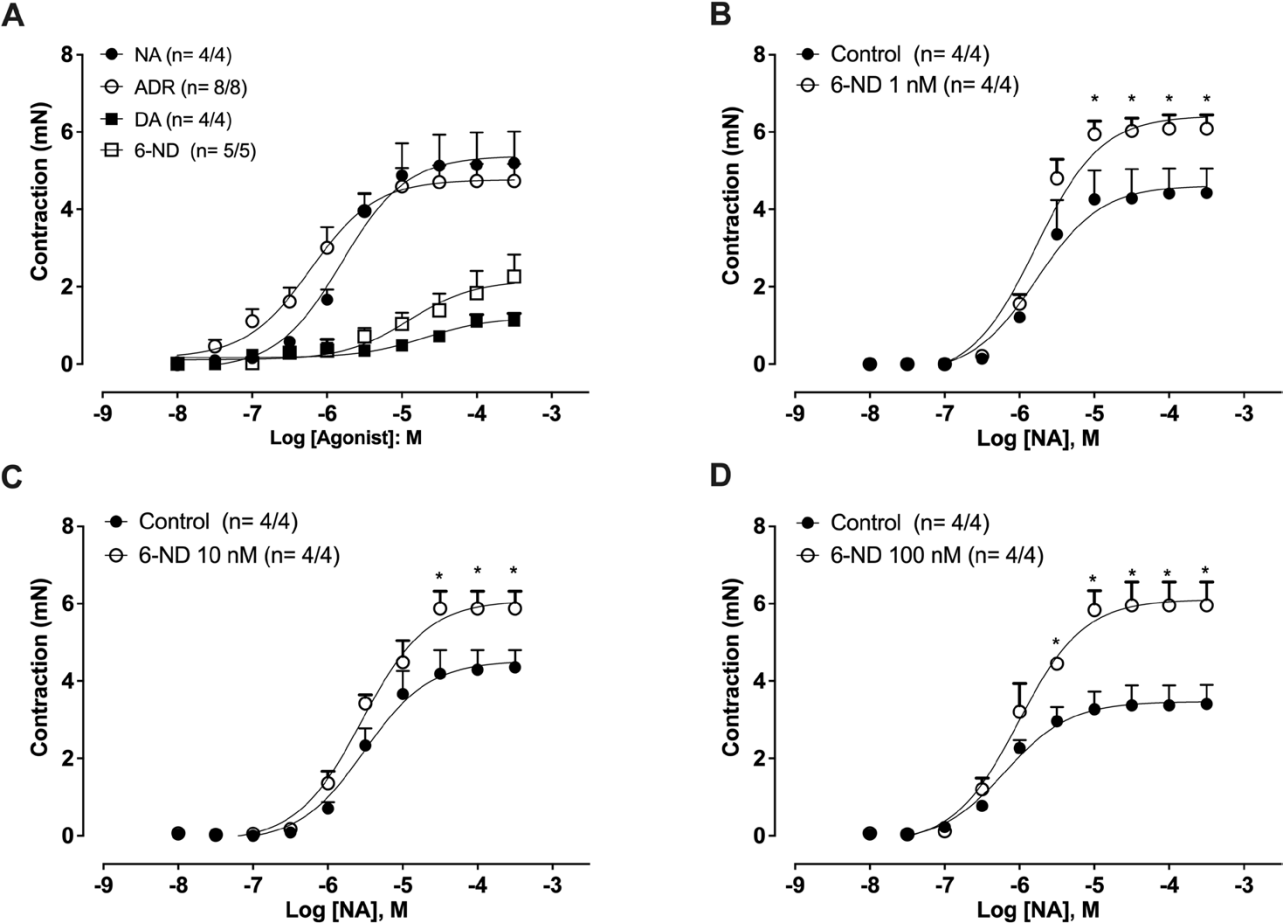
Concentration‐response curves for 6‐nitrodopamine, noradrenaline, adrenaline, and dopamine in mouse isolated seminal vesicles. 6‐Nitrodopamine (6‐ND), dopamine (DA), noradrenaline (NA), and adrenaline (ADR) induced concentration‐dependent contractions (A). Pre‐incubation (30 min) of the preparations with 6‐ND at 1 nM (B), 10 nM (C), or 100 nM (D) significantly increased the noradrenaline‐induced contractions. The number of seminal vesicles analyzed is expressed as *x*/*y*, where *x* is the number of animals and *y* is the number of seminal vesicles. *p < 0.05 compared with the respective concentration in the control group.

**FIGURE 7:**
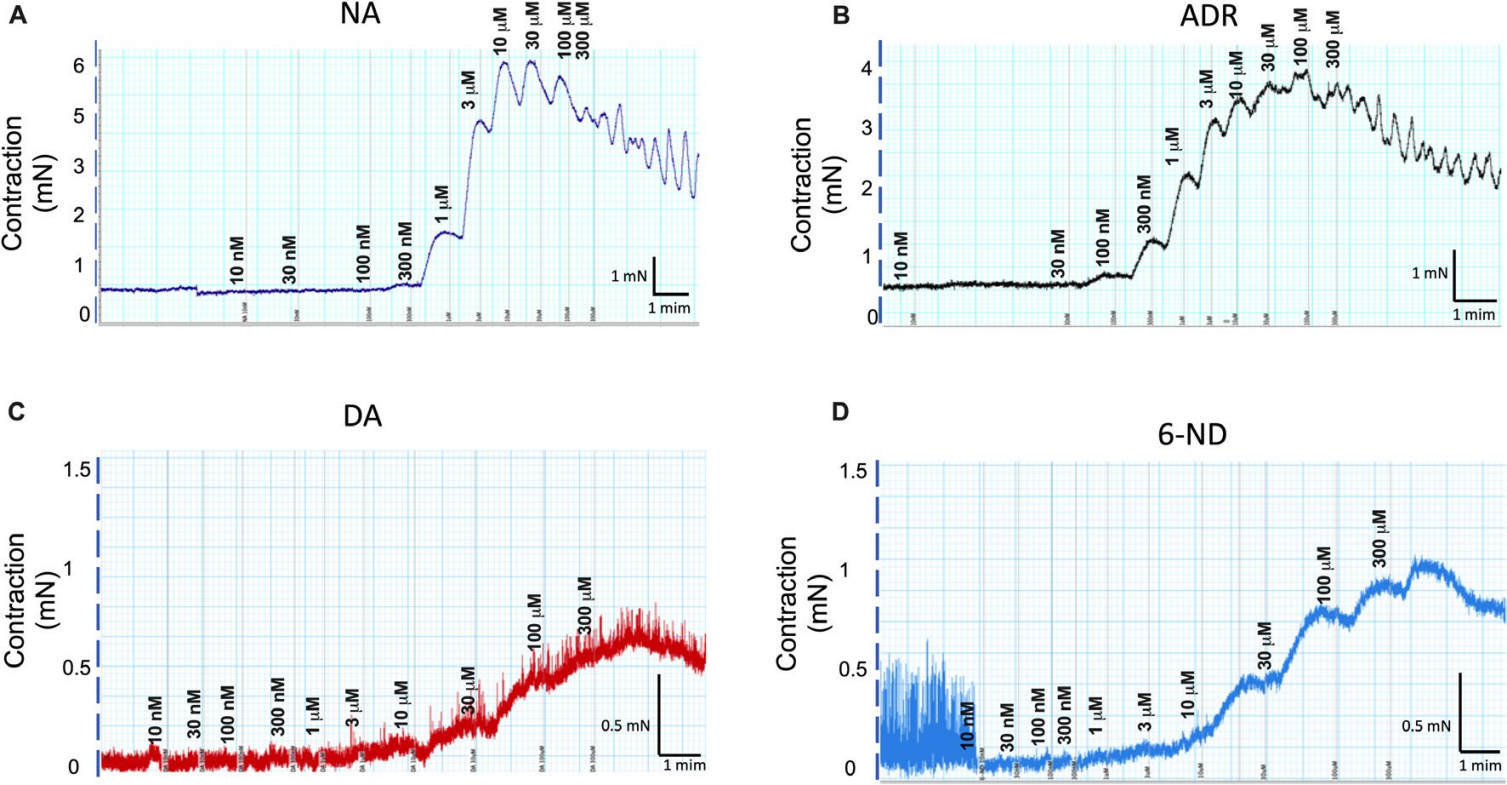
Representative traces of the concentration‐response curves for noradrenaline (NA; A), adrenaline (ADR; B), dopamine (DA; C), and 6‐nitrodopamine (6‐ND; D) in mouse isolated seminal vesicles.

6‐Nitrodopamine (10 nM–1 mM), noradrenaline (10 nM–300 μM), adrenaline (10 nM–300 μM), and dopamine (10 nM–1 mM) caused concentration‐dependent seminal vesicle contractions (Figure 6A). The pEC_50_ values for 6‐ND (4.90 ± 0.26, *n* = 5) are significantly lower than those for noradrenaline and adrenaline (5.80 ± 0.15, *n* = 4; and 6.22 ± 0.27, respectively; *n* = 8). However, 6‐ND exhibited a higher pEC_50_ than dopamine (4.86 ± 0.26; *n* = 4). The *E*_max_ values of 6‐ND and dopamine are lower than those of noradrenaline and adrenaline (2.3 ± 0.5, 1.2 ± 0.22, 5.2 ± 0.8 and 4.7 ± 0.4 mN for 6‐ND, dopamine, noradrenaline and adrenaline, respectively; *p* < 0.05). In addition, pre‐incubation of the mouse isolated seminal vesicle strips with 6‐ND at 1 nM (Figure 6B), 10 nM (Figure 6C), and 100 nM (Figure 6D) significantly potentiated the contractions induced by noradrenaline. Representative traces for the contractions induced by noradrenaline, adrenaline, dopamine, and 6‐ND are presented in Figure 7A–D, respectively.

### 3.7 Immunohistochemistry and FISH Assays for TH and eNOS in Mouse Seminal Vesicle

**FIGURE 8:**
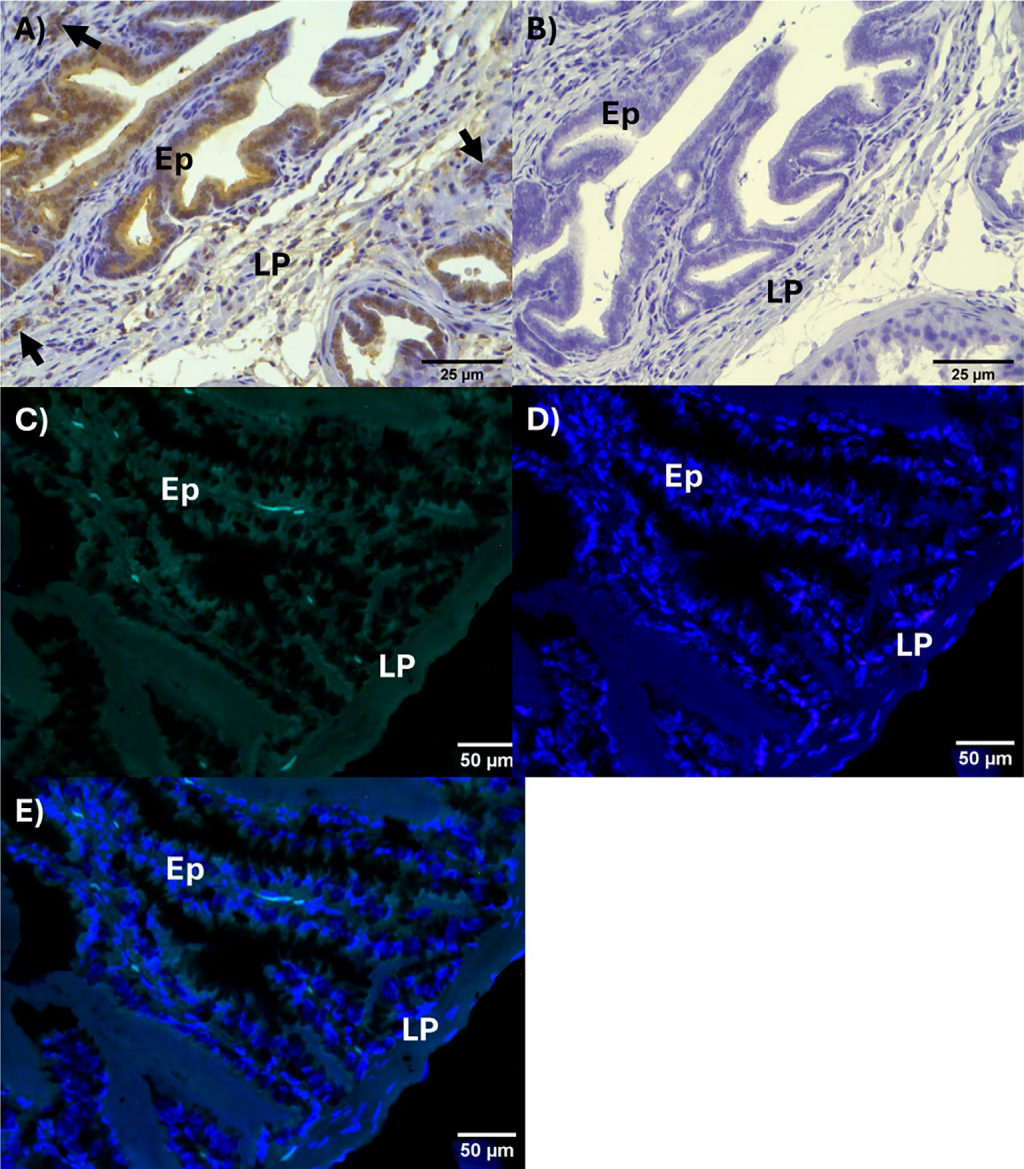
Illustrative photomicrographs of tyrosine hydroxylase (TH) immunoexpression in mouse seminal vesicle: Mucosae (epithelium + lamina propria). (A) TH immunostaining was detected in epithelial cells of the mouse seminal vesicle. The positivity was moderate to strong in epithelial cells (Ep) and endothelia of small vessels (arrows). (B) Negative control (omission of primary antibody). No positive or background staining; DAB/Hematoxylin, 200× (original magnification). (C–E) Detection of TH messenger RNA (mRNA) by fluorescence in situ hybridization (FISH) in mouse seminal vesicle. (C) TH mRNA, weak positivity in green (6‐FAM); (B) nuclear marker in blue (DAPI); (C) overlapping images (“overlay”). 6‐FAM and/or DAPI. The autofluorescence in red blood cells did not interfere with the interpretation of sections. 400× (original magnification). Ep, epithelium; LP, lamina propria.

Figure 8 illustrates the results of the immunohistochemical detection of TH in mouse seminal vesicle. Briefly, TH was positivity‐moderate to strong/diffuse in epithelium and vascular endothelium (Figure 8A). The negative control (omission of primary antibody) had no background staining (Figure 8B). TH expression was confirmed by the FISH assay, as illustrated in Figure 8C–E.

**FIGURE 9:**
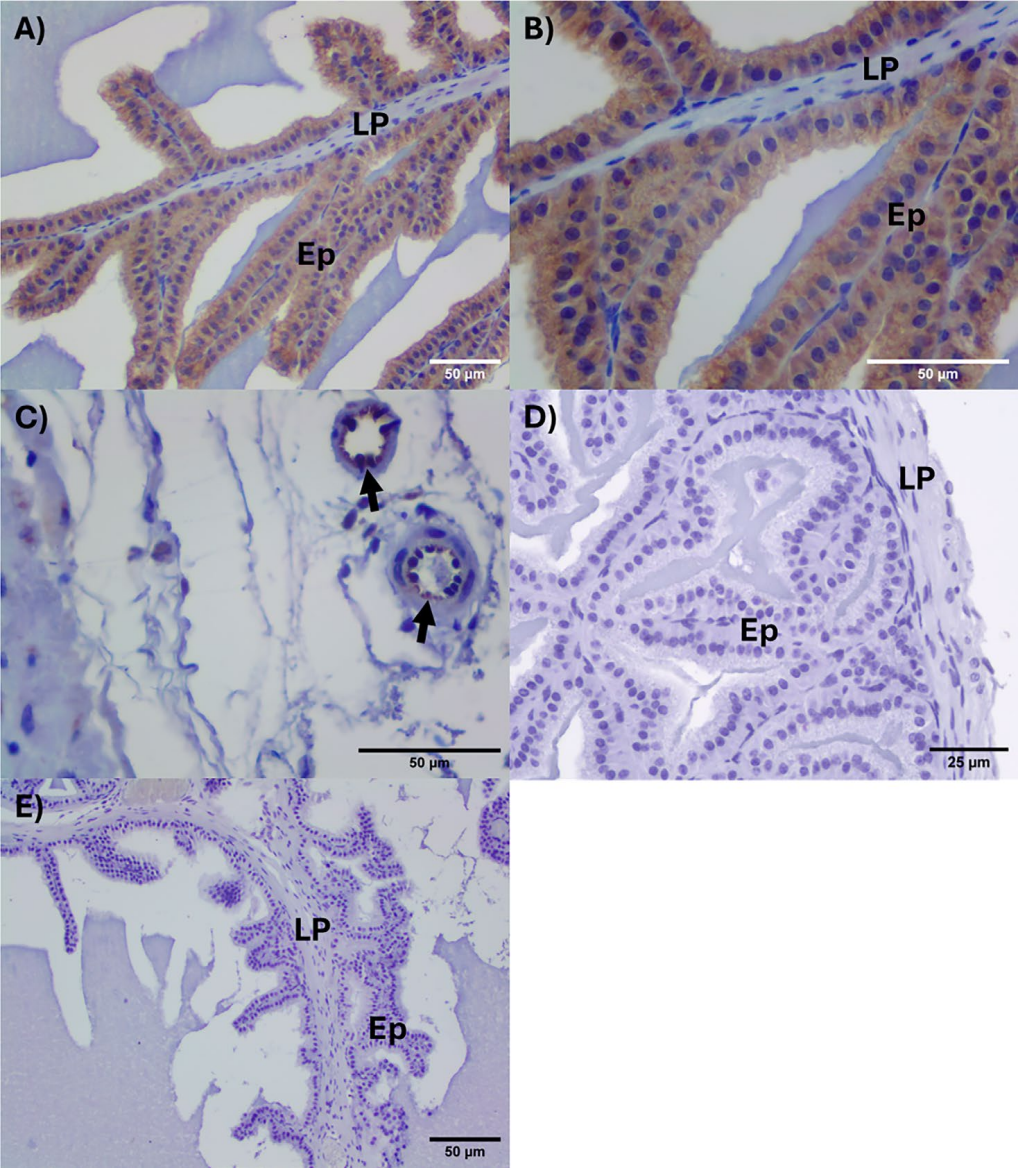
Endothelial nitric oxide synthase (eNOS) immunodetection in the mouse seminal vesicle. (A, B) Illustrate the moderate positivity for eNOS in epithelial cells (Ep); endothelial cells were also positive for eNOS as illustrated in (C, black arrows); (D) eNOS is negative in knockout (KO) mouse seminal vesicle; (E) negative control (omission of primary antibody). Immunoperoxidase (A, D) 200×; (B, C) 400×; (E) 100× (original magnification). Ep, epithelium; LP, lamina propria.

Endothelial nitric oxide synthase (eNOS) was detected by immunohistochemistry in the cytoplasm of epithelial cells, and the positivity intensity varied from weak to moderate (Figure 9A,B). eNOS positivity was also detected in the endothelium of some vessels of tunica adventitia, as illustrated in Figure 9C. Endothelial nitric oxide synthase was undetected in the seminal vesicles obtained from e/n/i NOS^−/−^ mice (Figure 9D). The negative control sections (omission of the primary antibody) were consistently devoid of any positivity (Figure 9E).

### 3.8 Western Blot Analysis of Phospho‐eNOS (Ser1177) in Mouse Seminal Vesicle

**FIGURE 10:**
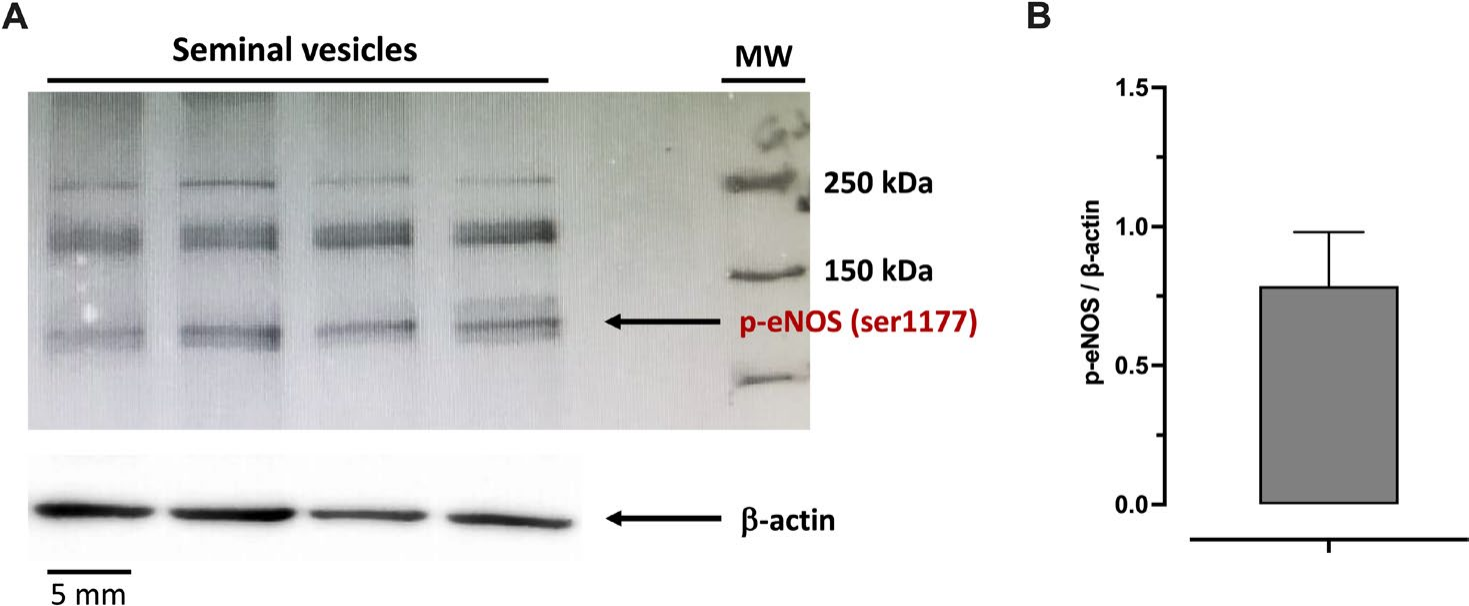
Representative immunoblot of phosphorylated eNOS at Ser1177 in seminal vesicles from control mice. (A) Shows the immunoblot of phosphorylated eNOS at Ser1177 (p‐eNOS Ser1177) in mouse isolated seminal vesicle samples (*n* = 4), using β‐actin as a loading control. (B) Shows the densitometric quantification of p‐eNOS expression normalized to β‐actin. Data are presented as mean ± SEM. MW, molecular weight markers.

Western blot analysis of seminal vesicle tissues from control mice revealed the presence of phosphorylated endothelial nitric oxide synthase (p‐eNOS) at Ser1177 (*n* = 4), as shown in Figure 10. The immunoblot showing p‐eNOS (Ser1177) expression, with β‐actin used as a loading control, is illustrated in Figure 10A. Densitometric quantification of p‐eNOS levels normalized to β‐actin is presented in Figure 10B.

## 4 Discussion

The results clearly indicate that 6‐ND is the major catecholamine released from mouse isolated seminal vesicles, which is consistent with human [18] and rat seminal vesicles [23]. Another important similarity across these species is that epithelial removal significantly decreased the 6‐ND release, indicating that the epithelium is the primary source for this catecholamine.

Cultured monkey amniotic epithelial cells (MAEC) express all the enzyme machinery necessary for catecholamine synthesis, including tyrosine hydroxylase, aromatic l‐amino acid decarboxylase, and dopamine‐β‐hydroxylase, as revealed by immunohistochemical staining [24]. Noradrenaline and dopamine have also been detected in extracts of cultured MAEC and human amniotic epithelial cells using HPLC coupled to electrochemical detection [25, 26]. Furthermore, human cultured retinal pigment epithelial cells synthesize and release dopamine when treated with high KCl solution (56 mM), as detected by HPLC [26]. The results here demonstrate by immunohistochemistry and fluorescence in situ hybridization that mouse seminal vesicle epithelium expresses tyrosine hydroxylase, as reported for both human [18] and rat [23] seminal vesicle, supporting the concept of epithelium‐derived catecholamines.

The use of NOS gene knockout mice in this study revealed new insights into 6‐ND biosynthesis by seminal vesicle epithelium. In contrast to findings from isolated mouse vas deferens, where basal 6‐ND release was significantly decreased in nNOS^−/−^ animals [5], suggesting involvement of nitrergic nerve terminals, the basal release of 6‐ND was reduced in seminal vesicles from eNOS^−/−^ animals, indicating that NO may also originate from the epithelium. Indeed, as demonstrated by immunohistochemistry, mouse seminal vesicle epithelium expresses eNOS, and the expression of phosphorylated eNOS by mouse seminal vesicle was confirmed by Western blot. Whether eNOS phosphorylation would be increased in stimulated seminal vesicles remains to be investigated.

It is interesting that NO is detectable in the exhaled breath, indicating that NO is produced by the human lung [27]. The three NOS isoforms have been identified by immunohistochemistry in the airway, with the epithelial cell‐derived iNOS considered the main responsible for the NO synthesis in normal human airways, since it is continuously expressed in airway epithelial cells [28]. Expression of iNOS is detected in rat lung epithelium [29] and bladder urothelium [20], and the presence of eNOS was demonstrated by immunohistochemistry in the rat seminal vesicle epithelium [23, 30].

Another interesting characteristic relies on the role of NOS in the 6‐ND biosynthesis. In rats pre‐treated with l‐NAME, the amount of 6‐nitro‐noradrenaline extracted from the brain was reduced by 50% [19]. The basal release of endothelium‐derived 6‐ND was significantly reduced, but not abolished, in vascular tissues treated with l‐NAME [1], suggesting either the existence of an NOS‐independent pathway for 6‐ND biosynthesis or an incomplete inhibition of NOS. Indeed, in mouse urinary bladder, the basal release of urothelium‐derived 6‐ND was inhibited by pre‐treatment with l‐NAME and abolished in triple NOS knockout mice [31]. However, the results reported here in mouse seminal vesicles from triple NOS knockout mice, where the basal release of 6‐ND was significantly reduced but not entirely abolished, and eNOS expression was undetected by immunohistochemistry, further support the possibility of an NOS‐independent pathway for 6‐ND biosynthesis. Whether this pathway involves the reduction of exogenous nitrites or involves another enzymatic pathway for NO synthesis remains to be determined.

Epithelium‐derived 6‐ND seems to play a major role in regulating seminal vesicle contractility. Although 6‐ND itself is a relatively weak contractile agonist, it significantly potentiates contractions induced by both noradrenaline and EFS. Seminal vesicle contractility is essential for emission during the ejaculatory process and involves both sympathetic and parasympathetic pathways [32]. Notably, noradrenaline is released from rat isolated seminal vesicles during EFS [33]. What mechanisms could underlie this observed potentiation? Pre‐incubation of human seminal vesicles with the adenylate cyclase activator forskolin (100 nM) results in a sevenfold increase in cAMP levels and inhibits EFS‐induced contractions [34], suggesting a modulatory role for adenylyl cyclase in seminal vesicle contractility. Moreover, incubation of human isolated seminal vesicles with either forskolin or the phosphodiesterase type 4 inhibitor rolipram [35] attenuates the contractions induced by both noradrenaline [36] and EFS [35]. In rat isolated atria, the positive chronotropic effects of the classical catecholamines noradrenaline, adrenaline, and dopamine, which are associated with adenylyl cyclase stimulation [1], are also strongly potentiated by 6‐ND [37]. The use of agarose coupled to 6‐ND for the purification of cardiomyocyte membranes revealed selective binding to three proteins that modulate adenylyl cyclase: cyclase‐associated protein 1 (CAP1), cyclase‐associated protein 2 (CAP2), and stromal interaction protein 1 (STIM1) [38]. CAP1 binds to and activates adenylyl cyclase in mammalian cells [39]. Ablation of CAP2 in mice results in dilated cardiomyopathy, accompanied by a significant reduction in heart rate [40]. STIM1 is expressed in cardiomyocytes [41], contains a single transmembrane domain [42], and is located in both the sarcoplasmic reticulum and plasma membrane [43]. STIM1 is also associated with adenylyl cyclase activation [44]. These proteins are compelling candidates for the proposed 6‐ND receptor‐mediated modulation of adenylyl cyclase in cardiomyocytes. The findings reported here suggest that in seminal vesicles the 6‐ND receptor may act as a negative modulator of adenylyl cyclase. Identifying the 6‐ND receptor in this tissue could clarify the mechanisms involved in the modulation of adrenergic stimulation by 6‐ND. Although there is no information on whether CAPs are expressed in mammalian seminal vesicles, adenylyl cyclase plays a major role in seminal vesicle contractility [45, 46].

The non‐selective α‐adrenoceptor antagonist phenoxybenzamine causes significant reduction of the rabbit seminal vesicle contractions induced by repetitive stimulation of the hypogastric nerve [13], whereas noradrenaline facilitates these contractions in the guinea‐pig seminal vesicles [13]. As previously mentioned, seminal vesicle contractility plays an important role in the emission phase of ejaculation, and ejaculatory disorders are the most common sexual dysfunctions in males [47]. The clinical use of α_1_‐adrenoceptor antagonists is associated with ejaculation disorders [48, 49], indicating that α‐adrenoceptors are important modulators of seminal vesicle [50]. Even though the investigation reported here is restricted to in vitro experiments, the finding that 6‐ND strongly potentiates the contractions induced by noradrenaline suggests that the synthesis/release of this novel endogenous epithelium‐derived catecholamine may play an important role in the ejaculatory process.

In conclusion, our data show that mouse seminal vesicles release epithelium‐derived 6‐ND by a mechanism involving eNOS activation. Quantification of phosphorylated eNOS in stimulated and non‐stimulated seminal vesicles should provide further information on the modulation of 6‐ND biosynthetic pathway. Altough 6‐ND itself behaves as a weak contractile agent of the seminal vesicle, but it markedly amplified the noradrenaline‐induced contractions; placing 6‐ND and the epithelium as novel endogenous modulators of seminal vesicle contractility.

## Author Contributions

Conceptualization: José Britto‐Júnior and Gilberto De Nucci. Data curation: José Britto‐Júnior and Gilberto De Nucci. Formal analysis: Gilberto De Nucci. Funding acquisition: Edson Antunes and Gilberto De Nucci. Investigation: José Britto‐Júnior, Pérola Rafaella Cedano Godoy, Denis Oliveira Lima, Felipe Fernandes Jacintho, Idam Hermawan, Hiroaki Shimokawa, Masato Tsutsui, Silvana Chiavegatto, Edson Antunes, and Gilberto De Nucci. Methodology: José Britto‐Júnior, Pérola Rafaella Cedano Godoy, Denis Oliveira Lima, Valéria B. de Souza, André A. Schenka, Felipe Fernandes Jacintho, Idam Hermawan, Hiroaki Shimokawa, Masato Tsutsui, Silvana Chiavegatto, Edson Antunes, and Gilberto De Nucci. Project administration: Gilberto De Nucci. Supervision: Edson Antunes. Visualization: Edson Antunes and Gilberto De Nucci. Writing – original draft: José Britto‐Júnior, Silvana Chiavegatto, Edson Antunes, and Gilberto De Nucci.

## Disclosure

The authors declare that all data were generated in‐house and that no paper mill was used.

## Ethics Statement

All experimental protocols were authorized by the Ethics Committee in Animal Use of UNICAMP (CEUA/UNICAMP, protocol numbers 5959‐1/2022 and 6337‐1/2023).

## Consent

The authors have nothing to report.

## Conflicts of Interest

The authors declare no conflicts of interest.

## Data Availability

The authors authorize the availability of any data used in this study.
